# Protein function annotation with Structurally Aligned Local Sites of Activity (SALSAs)

**DOI:** 10.1186/1471-2105-14-S3-S13

**Published:** 2013-02-28

**Authors:** Zhouxi Wang, Pengcheng Yin, Joslynn S Lee, Ramya Parasuram, Srinivas Somarowthu, Mary Jo Ondrechen

**Affiliations:** 1 Department of Chemistry and Chemical Biology, Northeastern University, Boston, MA 02115 USA; 2 Current address: Department of Molecular, Cellular, and Developmental Biology, 219 Prospect Street, Kline Biology Tower Room 826, Yale University, New Haven, CT 06520-8103 USA

## Abstract

**Background:**

The prediction of biochemical function from the 3D structure of a protein has proved to be much more difficult than was originally foreseen. A reliable method to test the likelihood of putative annotations and to predict function from structure would add tremendous value to structural genomics data. We report on a new method, Structurally Aligned Local Sites of Activity (SALSA), for the prediction of biochemical function based on a local structural match at the predicted catalytic or binding site.

**Results:**

Implementation of the SALSA method is described. For the structural genomics protein PY01515 (PDB ID 2aqw) from *Plasmodium yoelii*, it is shown that the putative annotation, Orotidine 5'-monophosphate decarboxylase (OMPDC), is most likely correct. SALSA analysis of YP_001304206.1 (PDB ID 3h3l), a putative sugar hydrolase from *Parabacteroides distasonis*, shows that its active site does not bear close resemblance to any previously characterized member of its superfamily, the Concanavalin A-like lectins/glucanases. It is noted that three residues in the active site of the thermophilic beta-1,4-xylanase from *Nonomuraea flexuosa *(PDB ID 1m4w), Y78, E87, and E176, overlap with POOL-predicted residues of similar type, Y168, D153, and E232, in YP_001304206.1. The substrate recognition regions of the two proteins are rather different, suggesting that YP_001304206.1 is a new functional type within the superfamily. A structural genomics protein from *Mycobacterium avium *(PDB ID 3q1t) has been reported to be an enoyl-CoA hydratase (ECH), but SALSA analysis shows a poor match between the predicted residues for the SG protein and those of known ECHs. A better local structural match is obtained with Anabaena beta-diketone hydrolase (ABDH), a known β-diketone hydrolase from *Cyanobacterium anabaena *(PDB ID 2j5s). This suggests that the reported ECH function of the SG protein is incorrect and that it is more likely a β-diketone hydrolase.

**Conclusions:**

A local site match provides a more compelling function prediction than that obtainable from a simple 3D structure match. The present method can confirm putative annotations, identify misannotation, and in some cases suggest a more probable annotation.

## Background

There are currently over 11,000 structural genomics (SG) protein structures in the Protein Data Bank (PDB) [1] and most of them are of unknown or uncertain function, as the inference of function from structure has proved to be more difficult than anticipated. Furthermore, when new structures of unknown function are determined, it is common practice to make a tentative functional assignment from the closest sequence match or the best 3D structure match to an annotated protein. Such tentative functional assignments are often incorrect [2]. Furthermore, one annotation error can propagate or "percolate" [2-4] in databases as additional proteins are annotated by automated or semi-automated means.

Overviews of current methods for the functional annotation of proteins from their sequence and/or structure have been given in recent reviews [5-8]. The simplest, and most commonly employed [6] methods seek the closest sequence matches using a search program such as BLAST [9], or alternatively the closest 3D structure match obtained from *e.g*. Dali [10], Combinatorial Extension (CE) [11], or Topofit [12], and then just transfer the function from the closest match to the query protein. However, even relatively high sequence similarity does not necessarily imply similar function [13]. Other types of sequence-based methods employ motif searching, phylogenetic profiling, or genome context. The Critical Assessment of Function Annotation (CAFA) experiment (http://biofunctionprediction.org/) seeks to assess the state of the current art of function prediction, chiefly from sequence. The aim of this work is to exploit structural information, together with computed chemical properties, to enhance function prediction capabilities.

It was hoped that SG would provide functional annotations for the protein products of newly-sequenced coding genes, as indeed the 3D structure can sometimes be indicative of function. Simple protein fold comparison does work in some cases, as domains having a common fold sometimes do have the same function. However, many folds have multiple functions. For instance, the Rossman fold and the TIM barrel each represent more than 50 different functions. The use of **local **3D structural motifs or templates, a feature of the present method, is now emerging as a more promising path for correct functional annotation from structure [14-19].

In spite of recent advances in protein function prediction, inference of biochemical function from the structure is difficult [20,21]. Hundreds of SG structures have no functional assignment at all and, for thousands of other SG proteins, functional hypotheses for SG proteins are putative and uncertain. Not all such hypotheses will prove in time to be correct, as examples below will illustrate. The ability to determine function from the 3D structure would add great value to this growing volume of SG data.

A different approach to functional annotation from 3D structure is presented here and is based on the combination of functional site prediction with local 3D structural alignment. Functional site predictions are obtained from Partial Order Optimum Likelihood (POOL) [22,23], a monotonicity-constrained maximum likelihood method, using computed chemical, electrostatic, and geometric properties, as well as phylogenetic information (if available), as input features. POOL places all of the residues in the input protein structure into an ordered list, ranked according to probability of participation in the active site. The top-ranked residues constitute the active site prediction. Structural alignments are obtained for sets of these local sites. Characteristic spatial patterns of predicted residues at the structurally aligned local sites of activity (SALSAs) are then used to identify specific types of biochemical function. The quality of the match of the predicted functional site in the query protein to functional sites in proteins of known function is measured using a scoring function. The present method can determine whether a putative functional assignment is likely to be correct or incorrect. In some cases where a protein is shown to be misannotated, a probable functional assignment is made.

## Methods

**Functional residue predictions **were made using POOL [22,23]. Input features for each residue in a given structure include: electrostatics information, as contained in THEMATICS metrics [24,25]; phylogenetic information from INTREPID [26,27]; and geometric information from ConCavity (structure only version) [28]. The top-ranked residues in the POOL output constitute the functional site prediction. Cut-off limits are specified for each case.

**Multiple structure alignments **are made for each set of proteins. The structural alignment of multiple structures of diverse function can be difficult and therefore multiple alignment methods [11,12,29] may be needed for some cases. In the examples shown here, T-Coffee [29] is used. For present purposes, a full alignment is not necessary. A quality alignment is only required in the local spatial region of the predicted active site.

**SALSA tables are constructed **for the locally aligned residues in the predicted active site. In a SALSA table, the rows represent individual protein structures and the columns represent spatially aligned positions.

**Consensus signatures **for a given functional subclass are established using POOL predictions on a set of previously characterized proteins with the same biochemical function, usually with common fold. To maximize sequence diversity in this reference set, sets of structures are sought with the lowest possible sequence identity among them. POOL-predicted residues of the same amino acid type in the same spatial position for the majority of the previously characterized proteins of common biochemical function then constitute the consensus signature for that functional group. The consensus signature for a given biochemical function thus consists of a series of amino acid types in specified spatial positions.

**SG proteins of unknown or uncertain function are analyzed **by POOL and the predictions are aligned with those of proteins of known function, or with the consensus signature.

**Scoring **the match between the predicted active site for the query protein and that of the consensus signature is performed using the BLOSUM62 matrix [30]. Scores are reported as a percentage of the maximum value (*i.e*. the score for the perfect match, the consensus signature with itself).

## Results and discussion

### Confirmation of annotation for PY01515, a putative Orotidine 5'-monophosphate decarboxylase (OMPDC)

Orotidine 5'-monophosphate decarboxylase (OMPDC) catalyzes one step in the pyrimidine biosynthesis pathway. It catalyzes the metal ion dependent decarboxylation of orotidine monophosphate (OMP) to uridine monophosphate (UMP) and CO_2 _[31,32]. OMPDC is a member of the ribulose phosphate binding barrel (RPBB) superfamily and has a TIM barrel [33] structure, with the active site located inside the beta barrel, spanning the eight beta strands. The structural genomics protein PY01515 (PDB ID 2aqw) is a putative OMPDC from *Plasmodium yoelii *[34].

The POOL-predicted functional site for PY01515 was aligned with eight different functional site types predicted by POOL for structures in the RPBB superfamily and a strong match was found with that of the OMPDCs and not with the other seven functional types. Five previously characterized OMPDC structures, those from *Bacillus subtilis *(PDB ID 1dbt), *Methanothermobacter thermautotrophicus *(PDB ID 1dvj), *Saccharomyces cerevisiae *(PDB ID 1dqw), *Escherichia coli *(PDB ID 1l2u), and *Plasmodium falciparum *(PDB ID 2za1), were used to establish the consensus signature of an OMPDC active site. These five previously characterized OMPDCs represent considerable sequence diversity, as shown in Table 1. With the exception of structures 1 and 4, which share sequence identity of 60%, all other pairs of structures have sequence identities in the 6% - 30% range.

**Table 1:** Sequence identity matrix for five previously characterized OMPDCs (structures 1-5) and the SG protein PY01515 (PDB ID 2aqw).

PDB ID:		1dbt	1dvj	1dqw	1l2u	2za1	2aqw
**1**	1dbt		0.240	0.260	0.600	0.060	0.240

**2**	1dvj	0.240		0.280	0.280	0.120	0.220

**3**	1dqw	0.260	0.280		0.300	0.080	0.280

**4**	1l2u	0.600	0.280	0.300		0.060	0.200

**5**	2za1	0.060	0.120	0.080	0.060		0.020

**6**	(SG) 2aqw	0.240	0.220	0.280	0.200	0.020	

For the five previously characterized OMPDCs, the important residues are predicted using the top 9% of the residues, as ranked by POOL, for each protein structure. When these five predicted active sites are structurally aligned, eight spatial positions are found to have common predicted residues across the five diverse, previously characterized OMPDCs. Table 2 shows this local structural alignment. The rows in Table 2 represent individual protein structures, with the five previously characterized OMPDCs listed first; the last row is the query protein from SG. The columns represent spatially coincident positions in the local structural alignment. The residues predicted by POOL are shown in uppercase; residues in lowercase are not in the top 9% of the POOL rankings. The previously reported catalytic residues [35,36] are shown in **boldface**. Positions 1-8 are positions in the consensus prediction, *i.e*. similar residues are predicted by POOL for the majority of the previously characterized OMPDCs. The row above each position gives the beta strand on which that position is located. For positions 1-5, 7, and 8, an identical residue is predicted by POOL for all five previously characterized OMPDCs. At position 6, a histidine is predicted for four out of the five previously characterized OMPDCs. For the *Plasmodium falciparum *structure, there is an asparagine, not predicted by POOL, at position 6. The consensus signature may be abbreviated as (D, K, D, K, D, H, P, R). The combination of residue types at the eight positions shown in Table 2 is unique to OMPDC within the RPBB superfamily. For instance, the lysine in position 2 and the proline in position 7 are not observed in the equivalent positions for any of the seven other functional subclasses of the RPBB superfamily.

**Table 2:** Local structural alignment of the consensus signature residues for the OMPDCs.

Structurally aligned signature active site residues for OMPDC									
		**β1**	**β2**	**β3**			**β4**	**β7**	**β8**

	PDB	1	2	3	4	5	6	7	8

	1dbt	D11	**K33**	**D60**	**K62**	**D65**	H88	P182	R215
	1dvj	D20	**K42**	**D70**	**K72**	**D75**	H98	P180	R203
Protein	1dqw	D37	**K59**	**D91**	**K93**	**D96**	H122	P202	R235
	1l2u	D22	**K44**	**D71**	**K73**	**D76**	H99	P189	R222
	2za1	D23	**K102 **	**D136**	**K138**	**D141**	n165	P264	R294

SG	2aqw	D23	K105	D139	K141	D144	n168	P267	R297

The first five rows represent previously-characterized OMPDCs. The sixth row is a putative OMPDC from Structural Genomics. The columns represent spatially coincident positions in the structural alignment; these positions are numbered 1-8. Known catalytic residues are shown in **boldface**. POOL-predicted residues are shown in uppercase; residues not predicted by POOL are shown in lowercase. The beta strand on which each position is located is given at the top of the column, above the position number. The good match between the SG protein and the known OMPDCs suggests common function.

The quality of a match with the consensus signature may be measured using a scoring matrix. Using the BLOSUM62 [30] matrix, the first four proteins listed in Table 2 have a score of 48 with the consensus signature; this score is 100% of the maximum value. The *Plasmodium falciparum *structure has a score of 39 (81% of the maximum value) against the consensus signature.

The structurally aligned residues for the SG protein PY01515 from *Plasmodium yoelii *are shown in the last row of Table 2. For seven out of the eight positions, POOL predicts residues that are identical to the consensus signature residues of the previously characterized OMPDCs. The only variation is in position 6, where there is an asparagine that is not predicted by POOL, just as in the *Plasmodium falciparum *OMPDC. PY01515 has a score of 39 (81% of the maximum value) against the consensus signature, using the BLOSUM62 scoring matrix. The strong match between the predicted active site for PY01515 and those of the previously characterized OMPDCs indicates that the putative OMPDC functional assignment is correct.

### YP_001304206.1 - a probable new functional type in the Concanavalin A-like lectins/glucanases superfamily

YP_001304206.1 (PDB ID 3h3l) is a putative sugar hydrolase from *Parabacteroides distasonis*, a commensal bacterium of the human intestinal tract. YP_001304206.1 is a member of the Concanavalin A-like lectins/glucanases superfamily.

**Figure 1:**
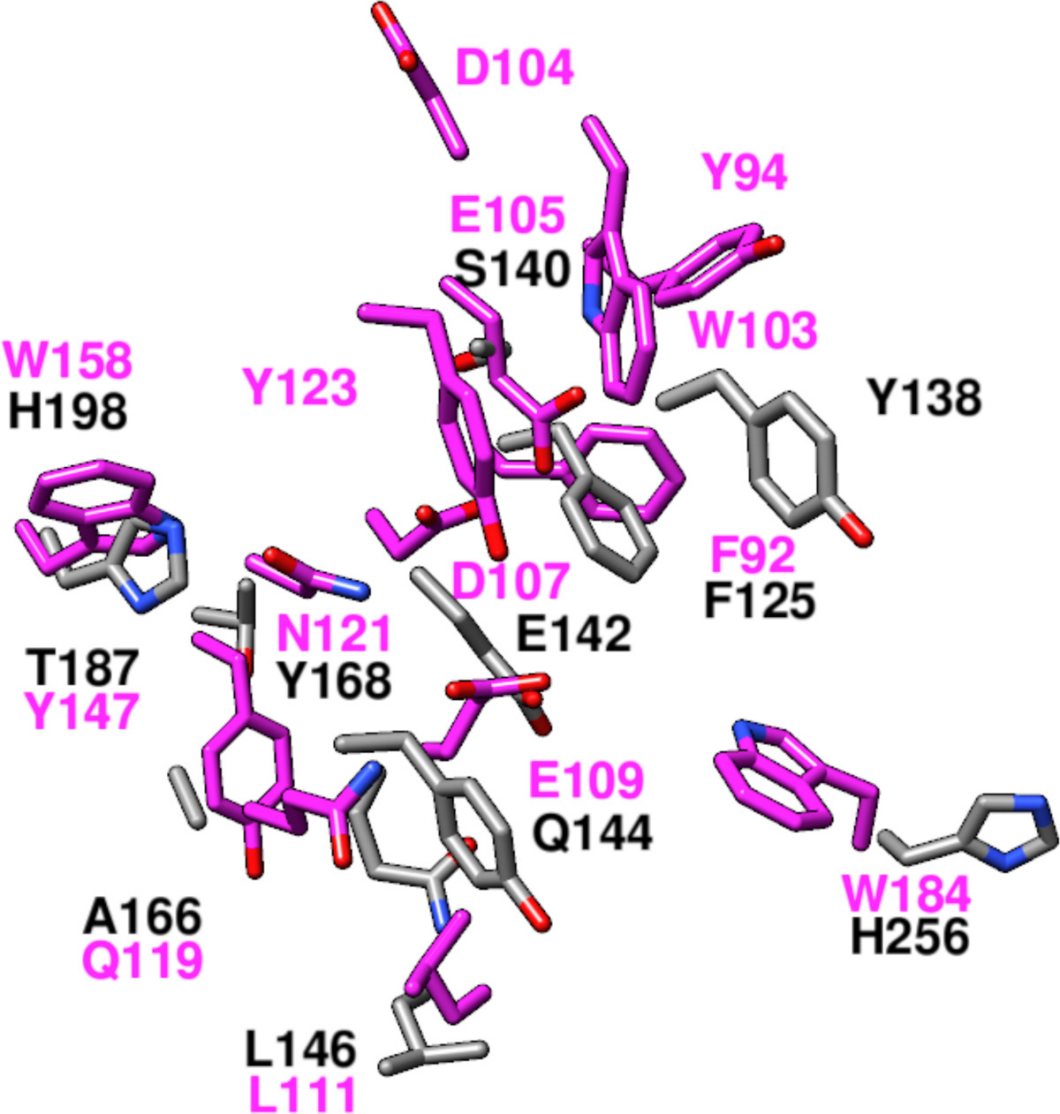
**Structural alignment of predicted residues for YP_001304206.1 (gray) with those of endo-1,3-1,4-beta-D-glucan 4-glucanohydrolase (pink)**.

The POOL-predicted functionally important residues for YP_001304206.1 show poor spatial overlap with those of all of the enzymes of known function within the Concanavalin A-like lectins/glucanases superfamily. Figure 1 shows a structural alignment of the predicted residues for YP_001304206.1 with those of its closest Dali [10,37] structural match, endo-1,3-1,4-beta-D-glucan 4-glucanohydrolase (PDB ID 2ayh), a representative member of the glycoside hydrolases family 16 (GH16). The residues for the query protein YP_001304206.1 are shown in gray and those for endo-1,3-1,4-beta-D-glucan 4-glucanohydrolase are shown in pink. Table 3 shows an alignment at the 14 consensus signature positions of GH16 for the representative GH16, endo-1,3-1,4-beta-D-glucan 4-glucanohydrolase, with the SG protein YP_001304206.1. Previously reported active site residues [38] are shown in **boldface**. POOL-predicted residues (top 8%) are shown in uppercase; residues not predicted are shown in lowercase. Note that the SG protein has a gap (no residue well aligned) at three of the consensus signature positions. For the alignment shown in Table 3, a negative BLOSUM62 score of -5 is obtained, corresponding to -5% of the maximum value of +97. The three catalytic residues for endo-1,3-1,4-beta-D-glucan 4-glucanohydrolase, E105, D107, and E109 [38], form an EXDXE motif on a common beta sheet and are seen forming a vertical line through the center of Figure 1. Note that these three residues overlap spatially in the alignment with S140, E142, and Q144 in YP_001304206.1. The very poor match score (negative) suggests that the function of endo-1,3-1,4-beta-D-glucan 4-glucanohydrolase cannot be transferred to YP_001304206.1.

**Table 3:** Local structural alignment of the residues in the GH16 consensus signature positions for the known representative GH16, endo-1,3-1,4-beta-D-glucan 4-glucanohydrolase, with the SG protein YP_001304206.1.

Spatial Positions→	1	2	3	4	5	6	7	8	9	10	11	12	13	14
2ayh (GH16)	F92	Y94	W103	d104	**E105**	**D107**	**E109**	L111	Q119	N121	Y123	Y147	W158	W184

3h3l (SG)	F125	-	Y138	-	s140	E142	q144	L146	A166	Y168	-	t187	H198	H256

Previously reported active site residues are shown in **boldface**. POOL-predicted residues (top 8%) are shown in uppercase; residues not predicted are shown in lowercase. The poor match suggests different functions.

**Figure 2:**
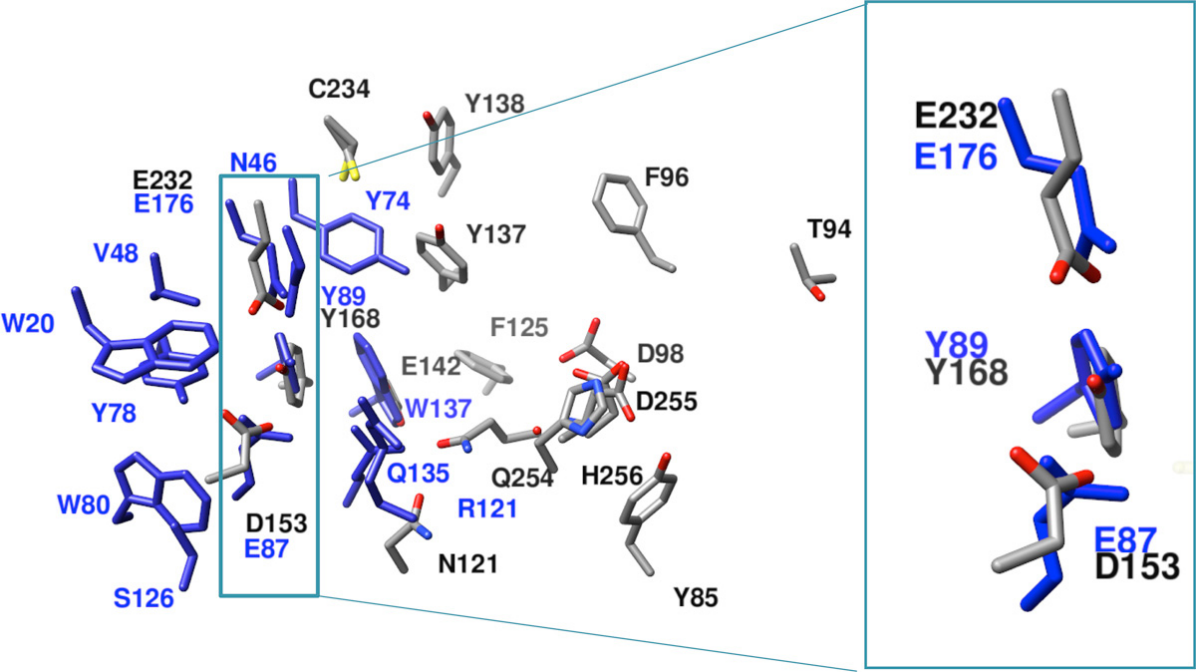
**Structural alignment of the POOL-predicted residues for the structural genomics protein YP_001304206.1 (gray) with those of a beta-1,4-xylanase from *Nonomuraea flexuosa *(blue)**. The overlap of the three catalytic residues, E87, Y89, and E176 of the xylanase with the aligned, predicted residues from YP_001304206.1 is highlighted in the blue box and shown in close-up in the large box on the right.

While the predicted active residues for YP_001304206.1 have low scores with those of the previously characterized members of the superfamily, one interesting comparison does emerge. The superposition of the predicted residues for the query protein with those of thermophilic beta-1,4-xylanase from *Nonomuraea flexuosa *(PDB ID 1m4w), a member of the xylanase/endoglucanase 11/12 family, shows some similarity in the catalytic residues. The reported active site residues [39] for thermophilic beta-1,4-xylanase from *Nonomuraea flexuosa *are Y78, E87, and E176. YP_001304206.1 possesses a spatially coincident triad in the local structural alignment consisting of the residues Y168, D153, and E232. This is illustrated in Figure 2, where the predicted residues for YP_001304206.1 (shown in gray) are structurally aligned with the predicted residues of the thermophilic beta-1,4-xylanase (shown in blue) from *Nonomuraea flexuosa*. The overlap of three of the predicted residues in the query protein, Y168, D153, and E232, with those of the catalytic residues of the xylanase, Y78, E87, and E176 is shown in the boxed region of Figure 2; a close-up of this region is shown in the large box on the right side of Figure 2. This suggests that the catalytic mechanism of the query protein may have similarities with that of the xylanase. However, as Figure 2 shows, the other residues, those involved in substrate recognition in the xylanase, are not very well conserved in YP_001304206.1. Furthermore, the predicted residues D98, D255, and H256 of YP_001304206.1, observed as a cluster in the center of Figure 2, appear to form a metal-binding motif that is not present in the xylanase. This suggests that YP_001304206.1 is a novel functional type in the Concanavalin A-like lectins/glucanases superfamily.

### An enoyl-CoA hydratase reported for *Mycobacterium avium *is incorrectly annotated

A structural genomics protein from *Mycobacterium avium *(PDB ID 3q1t), a potential target for the treatment of infectious disease, has been reported to be an enoyl-CoA hydratase (ECH). This SG protein and the ECHs are members of the ClpP/crotonase superfamily. The consensus signature residues for previously characterized ECHs were established using POOL predictions and SALSA. These residues, the spatial signature of an ECH catalytic site, are located in nine positions in the structural alignment. Then, the residues in the consensus signature were structurally aligned with residues in the SG *M. avium *structure. An alignment of the consensus signature residues, represented by enoyl-CoA hydratase from *Rattus norvegicus *(PDB ID 1ey3), with the corresponding spatially overlapping residues of the query protein, is shown in Table 4. Again, the rows represent individual protein structures and the columns represent spatial positions in the alignment. The known catalytic residues, A98, G141, E144, and E164 [40,41], are shown in **boldface**. Residues predicted by POOL are shown in uppercase and residues not predicted are shown in lowercase. The BLOSUM62 score between the SG protein and the known ECH is only 11, or 22% of the maximum value of 51, for these nine positions. Note further that the SG protein is missing the catalytic residues that correspond to E144 and E164 in the *Rattus norvegicus *ECH structure. These results strongly suggest that the reported enoyl-CoA hydratase annotation is incorrect.

**Table 4:** Local structural alignment of the predicted active site residues by SALSA for a known ECH from *Rattus norvegicus *(PDB ID 1ey3) with predicted residues for a Structural Genomics protein from *Mycobacterium avium *(PDB ID 3q1t), reported to be an ECH.

Spatial Positions→	1	2	3	4	5	6	7	8	9
Known ECH (1ey3)	**A98**	**g141**	**E144**	C149	D150	**E164**	R178	k241	N245

SG protein "ECH" (3q1t)	G76	A123	V126	a131	D132	H146	C160	k223	n227

Known catalytic residues are shown in **boldface**. Residues predicted by POOL are in uppercase; residues not predicted are in lowercase. Note the poor match between the residues of the SG protein with those of the representative ECH.

Comparison of the local site prediction for the SG protein with those of other members of the ClpP/crotonase superfamily reveals a much better match with ABDH (Anabaena beta diketone hydrolase), a known β-diketone hydrolase from *Cyanobacterium anabaena *(PDB ID 2j5s). The local alignment of the top POOL-predicted residues for the *M. avium *structure with residues from ABDH is shown in Table 5. The known catalytic residues for ABDH [42] are shown in **boldface**. Again, the columns represent overlapping spatial positions, but in Table 5 they are listed in order of the POOL rank for the *M. avium *structure (D155 is ranked first, H146 second, E244 third, ...). Thus all of the residues listed for the SG protein in Table 5 are predicted by POOL. Residues not predicted by POOL for ABDH are shown in lowercase. Notice that four of the top-ranked POOL residues for the SG protein are aligned with the known catalytic residues of ABDH: D153, H144, E243, and H43. The BLOSUM62 score between the SG protein and the known ABDH for these seven positions is 30, or 60% of the maximum value. These results suggest that the *M. avium *structure may be a β-diketone hydrolase, but perhaps with a native substrate different from that of the *Cyanobacterium anabaena *protein.

**Table 5:** Local structural alignment of the predicted residues for the SG protein from *Mycobacterium avium *(PDB ID 3q1t) with the corresponding residues of ABDH from *Cyanobacterium anabaena*.

POOL Ranking→	1	2	3	4	5	6	7
SG protein "ECH" (3q1t)	D155	H146	E244	D144	H44	C160	H156

Known ABDH (2j5s)	**D153**	**H144**	**E243**	D141	**H43**	l158	g154

The spatial positions 1 through 7 correspond to the ordinal values for the top seven residues in the POOL rank order for 3q1t.

Known catalytic residues for ABDH are shown in **boldface**. Residues predicted by POOL are in uppercase; residues not predicted are in lowercase. Note that the match between the residues of the SG protein and of ABDH is better than that of Table 4.

**Figure 3:**
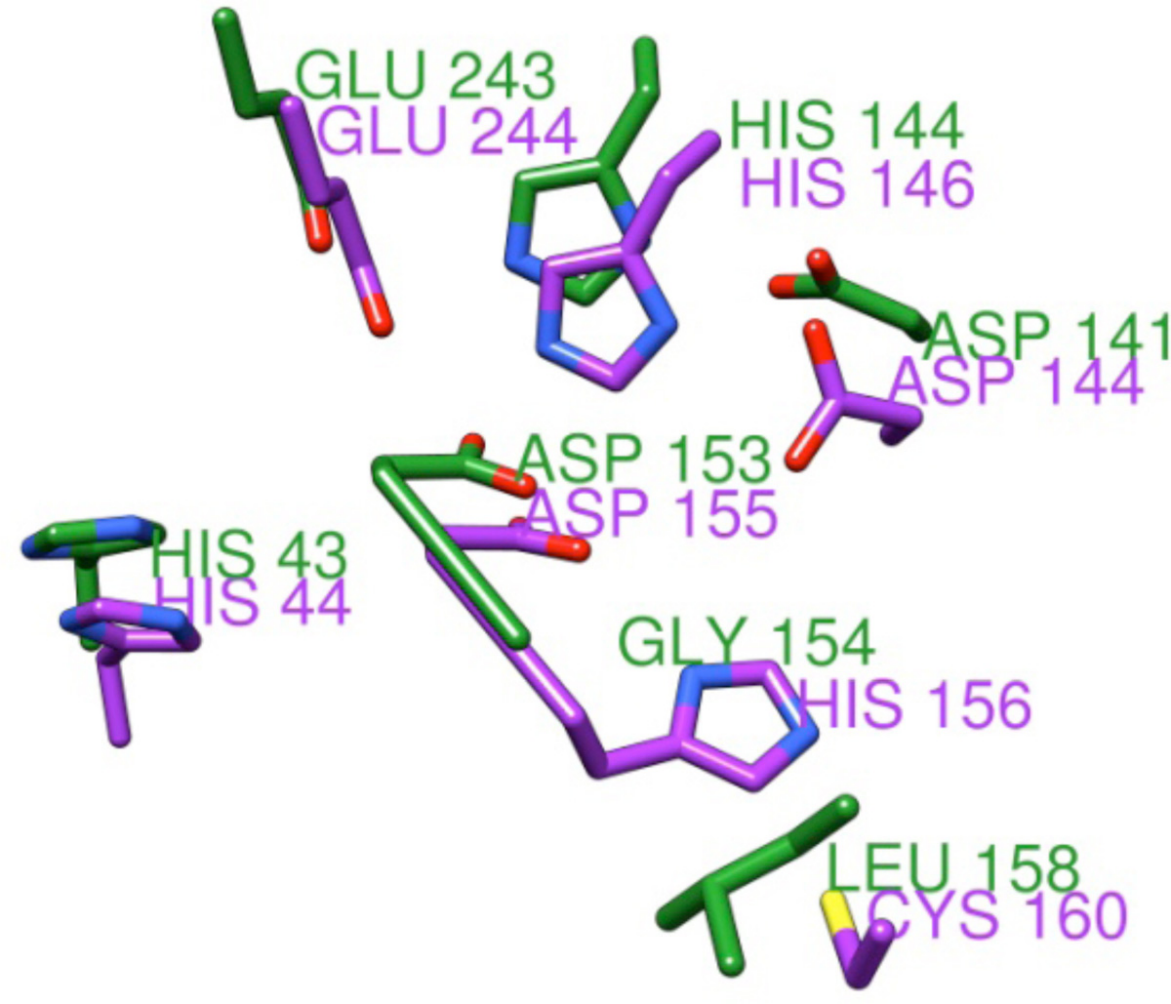
**Structural alignment of the top POOL-predicted residues for the SG protein (purple; PDB **3q1t**), reported to be an enoyl-CoA hydratase, with those of ABDH (green)**. H43, H144, D153, and E243 are known catalytic residues in ABDH.

Figure 3 illustrates the structural alignment of the top POOL-predicted residues for the SG *M. avium *structure (purple) with the corresponding residues from ABDH (green), showing that the known catalytic residues of ABDH have strong overlap with the top POOL-predicted residues for the SG protein.

## Conclusions

Local structural matching, as implemented by the SALSA method, provides a more compelling prediction of biochemical function than a simple, global 3D structure match. SALSA can confirm putative annotations, identify misannotations, suggest correct annotations, and, in some cases of misannotation, predict a more probable functional annotation.

For any given protein structure of previously characterized function, the list of residues reported in the literature to be important for the biochemical function is a subset of the list of residues predicted by POOL. This longer list is a key advantage of the present method, as it enables better discrimination between the functional subclasses.

To date, one prediction made by local site matching using our electrostatics-based functional site prediction has been verified experimentally by direct biochemical assays [43]. Further experimental testing of SALSA function predictions is in progress.

The BLOSUM62 scoring matrix has been used to measure the quality of the match between two predicted active sites. Whether there exists a better scoring matrix for this purpose is currently under investigation. At the present time, there are too few SG proteins with experimentally verified biochemical function to be able to translate the match score into a confidence metric, but as experimental testing progresses, this will become possible.

The SALSA method is amenable to automation and could be used to complement sequence-based function annotation methods, such as those evaluated in the CAFA experiments.

## Abbreviations

ABDH: Anabaena beta-diketone hydrolase; BLOSUM: BLOcks of amino acid SUbstitution Matrix; CAFA: Critical Assessment of Function Annotation; CE: Combinatorial Extension; ECH: enoyl-CoA hydratase; GH16: glycoside hydrolase family 16; INTREPID: INformation-theoretic TREe traversal for Protein functional site Identification; OMP: orotidine monophosphate; OMPDC: orotidine 5';-monophosphate decarboxylase; PDB: Protein Data Bank; POOL: Partial Order Optimum Likelihood; RPBB: Ribulose Phosphate Binding Barrel; SALSA: Structurally Aligned Local Sites of Activity; SG: Structural Genomics; THEMATICS: THEoretical Microscopic Anomalous TItration Curve Shapes; UMP: uridine monophosphate.

## Competing interests

The authors declare that they have no competing interests.

## Authors' contributions

All six authors performed the calculations, participated in the development of the methodology, and contributed to the writing of the manuscript. ZW had primary responsibility for the analysis of the Concanavalin A-like lectins/glucanases, PY for the *Mycobacterium avium *SG protein, and JSL for the OMPDCs. ZW, PY, and JSL contributed equally to this work.

## Author information

ZW, PY, JSL, and RP are doctoral candidates in the Department of Chemistry and Chemical Biology at Northeastern University. SS earned the Ph.D. degree in Chemistry from Northeastern University in 2011 and is currently engaged in postdoctoral research at Yale University. MJO is Professor of Chemistry and Chemical Biology and is Principal Investigator of the Computational Biology Research Group at Northeastern University.

## Declarations

This article has been published as part of *BMC Bioinformatics *Volume 14 Supplement 3, 2013: Proceedings of Automated Function Prediction SIG 2011 featuring the CAFA Challenge: Critical Assessment of Function Annotations. The full contents of the supplement are available online at http://www.biomedcentral.com/bmcbioinformatics/supplements/14/S3.
